# Physician Empathy and Chronic Pain Outcomes

**DOI:** 10.1001/jamanetworkopen.2024.6026

**Published:** 2024-04-11

**Authors:** John C. Licciardone, Yen Tran, Khang Ngo, David Toledo, Navya Peddireddy, Subhash Aryal

**Affiliations:** 1 Department of Family Medicine, University of North Texas Health Science Center at Fort Worth, Fort Worth; 2 University of North Texas Health Science Center at Fort Worth, Fort Worth; 3 School of Nursing, Johns Hopkins University, Baltimore, Maryland

## Abstract

**Importance:**

Empathy is an aspect of the patient-physician relationship that may be particularly important in patients with chronic pain.

**Objective:**

To measure the association of physician empathy with pain, function, and health-related quality of life (HRQOL) among patients with chronic low back pain.

**Design, Setting, and Participants:**

This cohort study included adult enrollees from the Pain Registry for Epidemiological, Clinical, and Interventional Studies and Innovation national pain research registry. Study dates were from April 1, 2016, to July 25, 2023, with up to 12 months of follow-up.

**Exposure:**

Physician empathy was assessed with the Consultation and Relational Empathy measure and dichotomized to yield very empathic physician and slightly empathic physician groups.

**Main Outcomes and Measures:**

Main outcomes were patient-reported pain, function, and HRQOL measured with a numerical rating scale for low back pain intensity, the Roland-Morris Disability Questionnaire for back-related disability, and the Patient-Reported Outcomes Measurement Information System for HRQOL deficits pertaining to anxiety, depression, fatigue, sleep disturbance, and pain interference. Data were collected at 5 quarterly encounters from registry enrollment through 12 months and analyzed with generalized estimating equations, including multivariable models to measure temporal trends and to adjust for baseline and longitudinal covariates.

**Results:**

Among the 1470 patients, the mean (SD) age was 53.1 (13.2) years, and 1093 (74.4%) were female. Patients completed 5943 encounters in which multivariable analyses demonstrated that greater physician empathy was inversely associated with pain intensity (β = −0.014; 95% CI, −0.022 to −0.006; *P* < .001), back-related disability (β = −0.062; 95% CI, −0.085 to −0.040; *P* < .001), and HRQOL deficits on each measure (eg, pain interference: β = −0.080; 95% CI, −0.111 to −0.049; *P* < .001). Correspondingly, compared with the slightly empathic physician group, the very empathic physician group reported lower mean pain intensity (6.3; 95% CI, 6.1-6.5 vs 6.7; 95% CI, 6.5-6.9; *P* < .001), less mean back-related disability (14.9; 95% CI, 14.2-15.6 vs 16.8; 95% CI, 16.0-17.6; *P* < .001), and fewer HRQOL deficits on each measure (eg, fatigue: 57.3; 95% CI, 56.1-58.5 vs 60.4; 95% CI, 59.0-61.7; *P* < .001). All physician empathy group differences were clinically relevant, with Cohen *d* statistics ranging from 0.21 for pain intensity to 0.30 for back-related disability, fatigue, and pain interference. Physician empathy was associated with more favorable outcomes than non-pharmacological treatments, opioid therapy, and lumbar spine surgery.

**Conclusions and Relevance:**

In this cohort study of adult patients with chronic pain, physician empathy was associated with better outcomes over 12 months. Greater efforts to cultivate and improve physician empathy appear warranted.

## Key Points

**Question:**

Is physician empathy associated with the outcomes of patients with chronic pain?

**Findings:**

In this cohort study that included 1470 adults with chronic low back pain, patients treated by very empathic physicians reported having significantly better and clinically relevant outcomes pertaining to pain, function, and health-related quality of life over 12 months compared with patients treated by slightly empathic physicians. Physician empathy was more strongly associated with favorable outcomes than were nonpharmacological treatments, opioid therapy, and lumbar spine surgery.

**Meaning:**

These findings suggest that physician empathy is an important aspect of the patient-physician relationship and was associated with better outcomes among patients with chronic pain.

## Teaser

This cohort study of adult patients with chronic pain assesses the association of physician empathy with patient outcomes, including patient-reported pain, function, and health-related quality of life.

## Introduction

The patient-physician relationship is fundamental to the practice of medicine. Although there is no agreement on how to define or study empathy,^1^ empathic opportunities arising during medical encounters may be missed, thereby posing a threat to the patient-physician relationship.^2^ Physician empathy may enhance patient adherence to treatment and improve clinical outcomes. A systematic review of randomized trials involving artificially manipulated practitioner empathy compared with usual care found modest patient benefits for several clinical conditions.^3^ Nevertheless, the review was limited by variability of interventions, practitioners, patients, and conditions, thereby yielding high statistical heterogeneity and low-quality evidence. Moreover, empathy outcomes were often assessed immediately after an encounter and never beyond 3 weeks.

Studies of physician empathy generally rely on observational research in a more natural setting. These involve physician self-assessed empathy or patient-perceived empathy. Unlike the latter, the former often measures physician attitudes about empathy rather than empathy itself.^4^ Because no correlation between physician and patient ratings of empathy has been observed, patients must be involved in assessing physician empathy.^5,6,7^ The Consultation and Relational Empathy (CARE) measure is the primary patient rating instrument for physician empathy,^7^ owing to its reliability^8^ and internal validity and consistency.^9^ The CARE measure may capture subtle nuances of patient interactions with physicians, thereby confirming its value in assessing relational components of empathy.^6^

The patient-physician relationship is vital among patients with chronic low back pain (CLBP) because patients often feel isolated, misunderstood, or stigmatized when an underlying cause of pain cannot be identified.^10^ A large correlation between physician empathy and satisfaction was reported immediately following a pain clinic consultation,^11^ and physician empathy was strongly associated with satisfaction among patients with CLBP even after controlling for confounders.^12^ A therapeutic alliance between such patients and physical therapists led to better pain and function outcomes over 8 weeks.^13^ Improved physician empathy over 3 months also was associated with better pain and health-related quality-of-life (HRQOL) outcomes among patients attending pain clinics.^14^ Because it is unclear whether these results would be sustained in general medical settings over time, we aimed to assess the association of patient-reported physician empathy with CLBP outcomes in such settings over 12 months.

## Methods

### Study Design and Patients

We conducted a cohort study using participants enrolled in the Pain Registry for Epidemiological, Clinical, and Interventional Studies and Innovation (PRECISION) from April 1, 2016, to July 25, 2023. The registry offers screening throughout the contiguous US using social media advertising.^15^ Those screened were eligible for the study if they were aged 21 to 79 years, had CLBP (≥3 months), had a physician who provided CLBP usual care, and had sufficient English-language proficiency to complete case report forms. Exclusion criteria were being pregnant or residing at an institutional facility. Hereinafter, the terms *participants* and *patients* may be used interchangeably to refer to registry participants who were patients of treating physicians in this study. Study participants provided self-reported data at registry enrollment and for up to 4 subsequent quarterly encounters over 12 months using a digital research platform for electronic data capture. This precluded missing item responses during completed encounters. Participants who missed consecutive encounters were considered lost to follow-up and disenrolled from the registry; however, available data were retained for analysis. To minimize potential objections to participation, the registry does not collect sensitive data such as income or health insurance coverage. It also does not collect names, demographic descriptors, or practice characteristics of physicians. Participants and, by extension, their physicians were blinded to research questions studied herein. This research was approved by the North Texas Institutional Review Board, and all participants provided written informed consent. This study followed the Strengthening the Reporting of Observational Studies in Epidemiology (STROBE) reporting guideline.^16^

### CARE Measure

Patients reported their physician’s empathy at enrollment using the CARE measure.^8,9^ It included 10 items about characteristics pertaining to physician empathy during medical encounters using an ordinal rating scale (score), including 1 for poor; 2, fair; 3, good; 4, very good; and 5, excellent. It was scored as the sum of responses to each item, ranging from 10 to 50, with higher scores indicating greater physician empathy. During development, the CARE measure demonstrated face and content validity and high internal validity (Cronbach α, 0.93), although scores were positively skewed (about one-fourth of patients reported a maximum physician empathy score).^8^ It was perceived by patients and physicians to be relevant to general medical encounters across different populations.^9^ Because CARE measure scores are positively skewed, we transformed them using a dichotomous variable theoretically based on its rating descriptors. Physicians whose scores were 30 or higher (ie, rated as good, very good, or excellent on most items) were classified as very empathic physicians (VEPs), whereas those whose scores were 29 or lower (ie, rated as poor or fair on most items) were classified as slightly empathic physicians (SEPs). The latter physicians made up approximately the lowest quartile of CARE measure scores. Data were analyzed only for encounters in which patients reported continuously having the same physician they had at enrollment. We assessed long-term stability of CARE measure scores among patients who retained the same physician 24 months later, when it was next administered.

### Outcome Measures

The outcomes were patient-reported pain, function, and HRQOL. Pain intensity was measured with a numerical rating scale for the typical pain level in the 7 days prior to each encounter, ranging from 0 to 10. Back-related disability, or function, on the encounter date was measured with the Roland-Morris Disability Questionnaire, and scores ranged from 0 to 24 to reflect difficulties that patients with CLBP may experience on each of its items.^17^ Because pain intensity may not adequately capture the overall experience of patients living with and negatively affected by CLBP,^18,19,20^ we assessed HRQOL deficits using the Patient-Reported Outcomes Measurement Information System with 29 items.^21^ This involved measures of anxiety, depression, fatigue, sleep disturbance, and pain interference. Each measure included 4 ordinal-scale items that were scored and normed according to the US general population to yield mean (SD) scores of 50 (10). The sole exception was sleep disturbance, which was normed using a calibration sample enriched with patients having chronic illness. Higher scores on all assessments represented worse outcomes for pain, function, and HRQOL.

### Baseline and Longitudinal Covariates

Comprehensive data pertaining to CLBP were collected at enrollment,^15^ and a series of baseline variables were selected to characterize patients and control for potential confounding. Sociodemographic characteristics included age, sex, race, ethnicity, and educational level. Health history included cigarette smoking status, musculoskeletal comorbidities (herniated disc, sciatica, osteoarthritis, and osteoporosis), and general medical comorbidities (hypertension, heart disease, diabetes, asthma, and depression). The CLBP history was characterized by ongoing duration (<1, 1-5, or >5 years). Data on CLBP treatment included nonpharmacological treatments ever used (exercise therapy, yoga, massage therapy, spinal manipulation, acupuncture, and cognitive behavioral therapy), opioid therapy, and lumbar spine surgery. Current opioid use and lumbar spine surgery were measured at each encounter. Race categories included American Indian or Alaska Native, Asian, Black or African American, Native Hawaiian or Other Pacific Islander, and White. (Racial categories other than White were combined in statistical analyses because small numbers in some racial categories yielded imprecise estimates that precluded meaningful interpretation.) Ethnicity categories included Hispanic and non-Hispanic. Categories were ascertained by self-report and were included in the study to serve as covariates used to adjust for potential confounding in the statistical analyses.

### Statistical Analysis

Patient characteristics according to physician empathy group were described and compared using number (percentage) or mean (SD). Generalized estimating equation (GEE) models were used initially to assess associations among CARE measure scores and each outcome over 12 months, including a time variable (number of quarterly intervals) to assess temporal trends. All GEE models used an autoregressive AR(1) correlation structure and fixed effects and included an empathy × time interaction term. For more tangible assessments of clinical relevance, GEE models also compared outcomes of the VEP vs SEP groups. The GEE analyses were repeated using a comprehensive multivariable model that included baseline and longitudinal data to adjust for potential confounding in addition to measuring time effects. Diagnostic plots, including normal Q-Q plots of Pearson residuals, supported appropriateness of GEE modeling and did not identify influential outliers. Clinically relevant differences in CARE measure scores and outcomes between VEP and SEP groups were assessed using thresholds for the magnitude of Cohen *d* (small effect: Cohen *d* = 0.2; medium effect: Cohen *d* = 0.5; and large effect: Cohen *d* = 0.8),^22^ with positive Cohen *d* statistics favoring the VEP group. Sensitivity analyses were performed by repeating the aforementioned analyses with alternative CARE measure score cut points to classify physician empathy.

Statistical power was estimated with the General Linear Mixed Model Power and Sample Size program for repeated measures designs^23^ and involved hypothesized mean differences in outcomes between VEP and SEP groups during 5 encounters over 12 months. Our sample size was sufficiently large to exceed 95% statistical power in detecting clinically important differences (Cohen *d* ≥ 0.2 in magnitude) between VEP and SEP groups on all outcomes in a wide variety of scenarios involving the correlation among outcome variables, base correlation of outcome variables at successive encounters within patients, and decay rate of the base correlation with increased time between encounters. The study was not designed to detect significant empathy × time interactions, owing to uncertainties about the nature of potential interactions (reversed, fully attenuated, or partially attenuated) and thresholds for clinical relevance.^24^ Data were managed and analyzed using SPSS Statistics, version 29 (IBM Inc). Hypotheses were assessed at the α level of .05 using 2-sided testing.

## Results

### Patient Characteristics

A total of 1470 patients were studied, including 1133 (77.1%) in the VEP group and 337 (22.9%) in the SEP group. The mean (SD) age of patients was 53.1 (13.2) years, 1093 (74.4%) were female, and 377 (25.6%) were male. The mean (SD) CARE measure score at enrollment was 38.4 (11.6). The mean (SD) CARE measure scores for the VEP and SEP groups were 43.7 (6.6) vs 20.6 (5.9) (*P* < .001; Cohen *d* = 3.57). The CARE measure scores were remarkably stable among 319 patients with 24-month follow-up (mean difference, −0.02; 95% CI, −1.32 to 1.29; *P* = .98; Cohen *d* = 0). The mean CARE measure score among 120 patients (8.2%) lost to follow-up was 38.5 (95% CI, 36.3-40.6) vs 38.4 (95% CI, 37.8-39.0) for the remaining patients (*P* = .95; Cohen *d* = 0.01). Baseline characteristics of the VEP and SEP groups were generally comparable, although there were marginally significant differences indicating that SEP group patients were more likely to be current smokers and to report a history of herniated disc, sciatica, and depression (Table 1).

**Table 1.** Baseline Patient Characteristics by Physician Type^a^

Characteristic	Physician type^b^		*P* value
	Very empathic (n = 1133)	Slightly empathic (n = 337)	
CARE measure score, mean (SD)	43.7 (6.6)	20.6 (5.9)	<.001
Age, mean (SD), y	53.4 (13.3)	52.0 (12.9)	.08
Sex			
Female	841 (74.2)	252 (74.8)	.84
Male	292 (25.8)	85 (25.2)	
Race			
American Indian or Alaska Native	15 (1.3)	5 (1.5)	.75
Asian	23 (2.0)	6 (1.8)	
Black or African American	198 (17.5)	51 (15.1)	
Native Hawaiian or Other Pacific Islander	3 (0.3)	2 (0.6)	
White	894 (78.9)	273 (81.0)	
Ethnicity			
Hispanic	99 (8.7)	32 (9.5)	.67
Non-Hispanic	1034 (91.3)	305 (90.5)	
Educational level, mean (SD)^c^	4.5 (2.0)	4.5 (1.8)	.89
Cigarette smoking status			
Never or former smoker	951 (83.9)	265 (78.6)	.02
Current smoker	182 (16.1)	72 (21.4)	
History of musculoskeletal comorbidities			
Herniated disc			
No	709 (62.6)	188 (55.8)	.02
Yes	424 (37.4)	149 (44.2)	
Sciatica			
No	597 (52.7)	157 (46.6)	.05
Yes	536 (47.3)	180 (53.4)	
Osteoarthritis			
No	635 (56.0)	171 (50.7)	.09
Yes	498 (44.0)	166 (49.3)	
Osteoporosis			
No	981 (86.6)	290 (86.1)	.80
Yes	152 (13.4)	47 (13.9)	
History of general medical comorbidities			
Hypertension			
No	632 (55.8)	205 (60.8)	.10
Yes	501 (44.2)	132 (39.2)	
Heart disease			
No	1011 (89.2)	303 (89.9)	.72
Yes	122 (10.8)	34 (10.1)	
Diabetes			
No	908 (80.1)	280 (83.1)	.23
Yes	225 (19.9)	57 (16.9)	
Asthma			
No	827 (73.0)	250 (74.2)	.66
Yes	306 (27.0)	87 (25.8)	
Depression			
No	509 (44.9)	129 (38.3)	.03
Yes	624 (55.1)	208 (61.7)	
No. of comorbidities, mean (SD)	3.0 (1.9)	3.1 (1.8)	.17
Duration of low back pain, y			
<1	79 (7.0)	24 (7.1)	.31
1-5	302 (26.7)	76 (22.6)	
>5	752 (66.4)	237 (70.3)	
Nonpharmacological treatments ever used for chronic low back pain			
Exercise therapy			
No	388 (34.2)	111 (32.9)	.66
Yes	745 (65.8)	226 (67.1)	
Yoga			
No	784 (69.2)	227 (67.4)	.52
Yes	349 (30.8)	110 (32.6)	
Massage therapy			
No	540 (47.7)	162 (48.1)	.89
Yes	593 (52.3)	175 (51.9)	
Spinal manipulation			
No	558 (49.2)	156 (46.3)	.34
Yes	575 (50.8)	181 (53.7)	
Acupuncture			
No	868 (76.6)	263 (78.0)	.58
Yes	265 (23.4)	74 (22.0)	
Cognitive behavioral therapy			
No	974 (86.0)	278 (82.5)	.12
Yes	159 (14.0)	59 (17.5)	
No. of nonpharmacological treatments ever used for chronic low back pain, mean (SD)	2.4 (1.6)	2.4 (1.6)	.43
Current opioid use for chronic low back pain			
No	759 (67.0)	239 (70.9)	.17
Yes	374 (33.0)	98 (29.1)	
Ever had lumbar spine surgery			
No	926 (81.7)	267 (79.2)	.30
Yes	207 (18.3)	70 (20.8)	

Abbreviation: CARE, Consultation and Relational Empathy.

^a^ Data are presented as number (percentage) unless otherwise indicated.

^b^ Physicians were classified as very empathic if their CARE measure scores were 30 or higher and as slightly empathic if their scores were 29 or lower. Scores ranged from 10 to 50, with higher scores indicating greater physician empathy. Slightly empathic physicians made up approximately the lowest quartile on the CARE measure.

^c^ Educational level was scored as 1 to indicate no high school diploma; 2, high school graduate or high school equivalency diploma; 3, some college, no degree; 4, occupational, technical, or vocational program; 5, associate’s degree; 6, bachelor’s degree; 7, master’s degree; or 8, professional school degree or doctoral degree.

### Longitudinal Outcomes of GEE Models Without Covariate Adjustment

Patients completed 5943 encounters, including 1470, 1296, 1192, 989, and 996 at successive encounters over 12 months. In GEE models that included only empathy, time, and empathy × time as explanatory variables for outcomes over 12 months, the CARE measure score was inversely associated with pain intensity (β = −0.013; 95% CI, −0.022 to −0.005; *P* = .003), back-related disability (β = −0.066; 95% CI, −0.091 to −0.040; *P* < .001), and each HRQOL deficit (eg, depression: β = −0.106; 95% CI, −0.150 to −0.062; *P* < .001) (eTable 1 in Supplement 1). Pain intensity decreased over time (β = −0.094; 95% CI, −0.185 to −0.003; *P* = .04), whereas back-related disability and HRQOL deficits did not change over time. There were generally no empathy × time interaction effects observed in these or subsequent analyses throughout the study.

**Figure 1.**
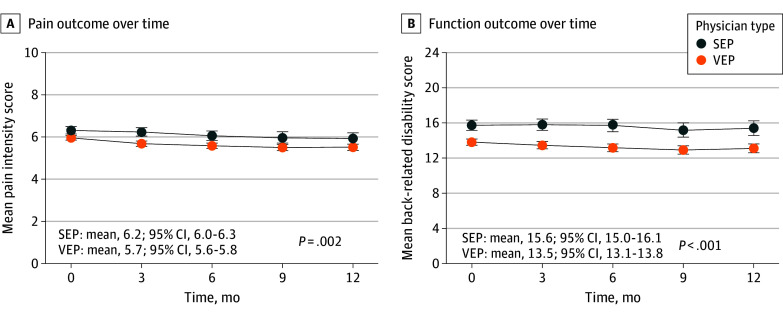
Pain and Function Outcomes Over Time A, Measured with a numerical rating scale for the typical pain level in the 7 days prior to each encounter, with scores ranging from 0 to 10. B, Measured with the Roland-Morris Disability Questionnaire, with scores ranging from 0 to 24. Higher scores on both assessments indicate worse outcomes for pain and function. Physicians were classified as very empathic if their Consultation and Relational Empathy (CARE) measure scores were 30 or higher and as slightly empathic if their scores were 29 or lower. Scores range from 10 to 50, with higher scores indicating greater physician empathy. Slightly empathic physicians made up approximately the lowest quartile on the CARE measure. Summary measures and *P* values are for the entire 12-month period adjusted for time and empathy × time interaction. Error bars represent 95% CIs. SEP indicates slightly empathic physician; VEP, very empathic physician.

**Figure 2.**
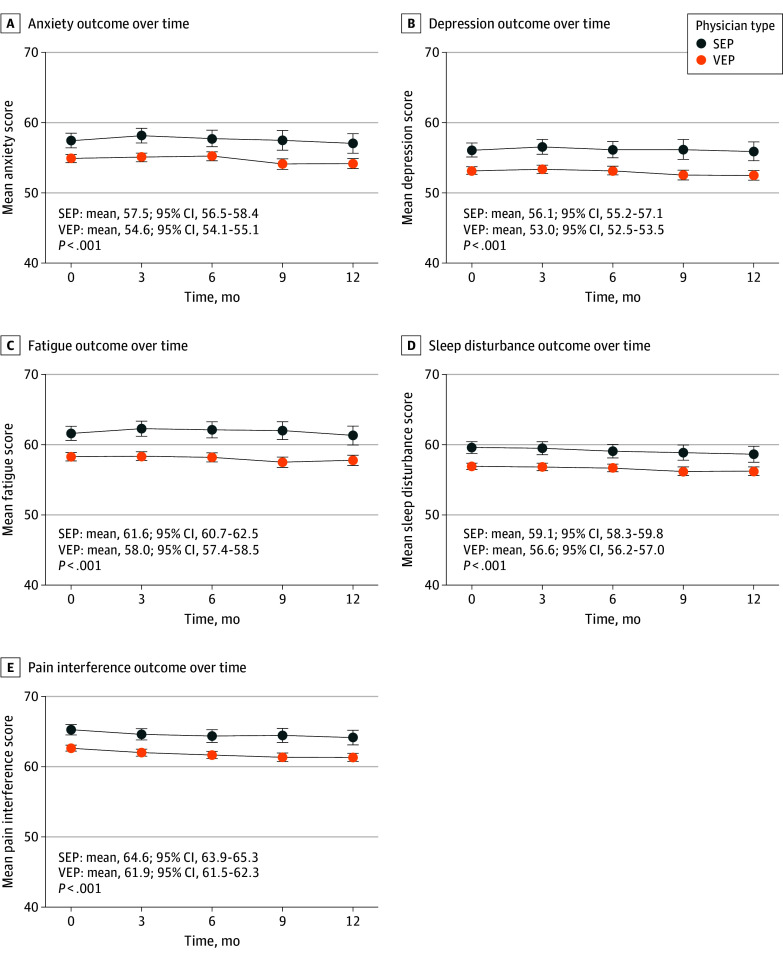
Health-Related Quality-of-Life Outcomes Over Time Health-related quality of life was measured using the Patient-Reported Outcomes Measurement Information System with 29 items. Each deficit measure included 4 ordinal-scale items that were scored and normed according to the US general population to yield mean (SD) scores of 50 (10). The sole exception was sleep disturbance, which was normed using a calibration sample enriched with patients having chronic illness. Higher scores indicate worse outcomes. Physicians were classified as very empathic if their Consultation and Relational Empathy (CARE) measure scores were 30 or higher and as slightly empathic if their scores were 29 or lower. Scores ranged from 10 to 50, with higher scores indicating greater physician empathy. Slightly empathic physicians made up approximately the lowest quartile on the CARE measure. Summary measures and *P* values are for the entire 12-month period adjusted for time and empathy × time interaction. Error bars represent 95% CIs. SEP indicates slightly empathic physician; VEP, very empathic physician.

Mean pain intensity in the VEP group was 5.7 (95% CI, 5.6-5.8) vs 6.2 (95% CI, 6.0-6.3) in the SEP group (*P* = .002) (Figure 1A). Similarly, mean back-related disability was 13.5 (95% CI, 13.1-13.8) in the VEP group vs 15.6 (95% CI, 15.0-16.1) in the SEP group (*P* < .001) (Figure 1B). Mean scores on each HRQOL deficit measure were also lower in the VEP group compared with the SEP group (Figure 2); the score for mean anxiety in the VEP group was 54.6 (95% CI, 54.1-55.1) vs 57.5 (95% CI, 56.5-58.4) in the SEP group (*P* < .001) and for sleep disturbance, 56.6 (95% CI, 56.2-57.0) in the VEP group vs 59.1 (95% CI, 58.3-59.8) in the SEP group (*P* < .001). All between-group differences pertaining to pain, function, and HRQOL yielded Cohen *d* statistics that met criteria for clinical relevance, ranging from 0.22 for pain intensity to 0.36 for fatigue (eTable 2 in Supplement 1).

### Longitudinal Outcomes of Comprehensive GEE Models With Covariate Adjustment

In GEE models that included empathy, time, empathy × time, and the full array of explanatory variables for outcomes over 12 months, the CARE measure score was inversely associated with pain intensity (β = −0.014; 95% CI, −0.022 to −0.006; *P* < .001), back-related disability (β =  −0.062; 95% CI, −0.085 to −0.040; *P* < .001), and each HRQOL deficit (eg, pain interference: β = −0.080; 95% CI, −0.111 to −0.049; *P* < .001) (eTable 3 in Supplement 1). Mean pain intensity in the VEP group was 6.3 (95% CI, 6.1-6.5) vs 6.7 (95% CI, 6.5-6.9) in the SEP group (*P* < .001) (eTable 4 in Supplement 1). Similarly, mean back-related disability was 14.9 (95% CI, 14.2-15.6) in the VEP group vs 16.8 (95% CI, 16.0-17.6) in the SEP group (*P* < .001). Mean scores on each HRQOL deficit measure were also lower in the VEP group compared with the SEP group (eg, fatigue: 57.3; 95% CI, 56.1-58.5 in the VEP group vs 60.4; 95% CI, 59.0-61.7 in the SEP group; *P* < .001). All between-group differences pertaining to pain, function, and HRQOL remained clinically relevant, with Cohen *d* statistics ranging from 0.21 for pain intensity to 0.30 for back-related disability, fatigue, and pain interference.

### Sensitivity Analyses for Alternative Classifications of Physician Empathy

Greater levels of physician empathy were generally associated with better outcomes in pain, function, and HRQOL in analyses that were unadjusted (eTable 5 in Supplement 1) and adjusted for multiple covariates (eTable 6 in Supplement 1) in addition to time. The favorability of having a more empathic physician generally increased as the CARE measure score cut point for greater vs lesser physician empathy decreased. The greatest group differences, which were observed for patients having physicians with CARE measure scores 20 or higher vs 19 or lower, involved Cohen *d* statistics ranging from 0.29 for pain intensity to 0.56 for fatigue (eTable 5 in Supplement 1) and from 0.24 for anxiety to 0.46 for fatigue (eTable 6 in Supplement 1).

### Clinical Relevance of Having a VEP

Although the outcomes of having a VEP exceeded the threshold for clinical relevance in models that were covariate unadjusted (eTable 2 in Supplement 1) and covariate adjusted (eTable 4 in Supplement 1), they were classified as small effects in the main analyses, and no empathy × time interaction effects were observed. However, the magnitude of physician empathy effects exceeded those reported for nonpharmacological treatments, current opioid use, and lumbar spine surgery, as demonstrated by their respective β coefficients (Table 2 and Table 3). Moreover, unlike physician empathy, patients receiving these 3 treatments generally reported worse outcomes. For example, current opioid use was associated with greater pain intensity (β = 0.351; 95% CI, 0.221-0.481; *P* < .001) and back-related disability (β = 1.205; 95% CI, 0.870-1.541; *P* < .001), and lumbar spine surgery was associated with greater back-related disability (β = 0.992; 95% CI, 0.344-1.639; *P* = .003) (Table 2). Furthermore, although not significant, empathy × time interaction effects trended to favor the VEP group for pain, function, and all HRQOL outcomes except sleep disturbance.

**Table 2.** Characteristics Associated with Pain and Function Outcomes Over Time^a^

Characteristic	Low back pain intensity, β (95% CI)^b^^,^^c^	*P* value	Back-related disability, β (95% CI)^c^^,^^d^	*P* value
Physician type^e^				
SEP (CARE measure score ≤29)	[Reference]	<.001	[Reference]	<.001
VEP (CARE measure score ≥30)	−0.387 (−0.605 to −0.168)		−1.732 (−2.353 to −1.111)	
Time (No. of quarterly intervals)	−0.080 (−0.134 to −0.026)	.003	−0.069 (−0.187 to 0.050)	.26
Physician empathy group × time^f^	−0.013 (−0.074 to 0.048)	.68	−0.065 (−0.204 to 0.075)	.36
Age, y^g^	0.007 (0 to 0.013)	.04	−0.005 (−0.027 to 0.016)	.62
Sex				
Female	0.218 (0.036 to 0.400)	.02	0.740 (0.121 to 1.359)	.02
Male	[Reference]		[Reference]	
Race				
Non-White^h^	0.935 (0.735 to 1.135)	<.001	1.519 (0.838 to 2.199)	<.001
White	[Reference]		[Reference]	
Ethnicity				
Hispanic	0.377 (0.107 to 0.648)	.006	0.473 (−0.505 to 1.451)	.34
Non-Hispanic	[Reference]		[Reference]	
Educational level^i^	−0.217 (−0.261 to −0.172)	<.001	−0.821 (−0.966 to −0.676)	<.001
Cigarette smoking status				
Never or former smoker	[Reference]	<.001	[Reference]	<.001
Current smoker	0.515 (0.302 to 0.729)		1.445 (0.740 to 2.151)	
Duration of low back pain, y				
<1	[Reference]	NA	[Reference]	NA
1-5	0.188 (−0.172 to 0.548)	.31	−0.090 (−1.282 to 1.102)	.88
>5	0.297 (−0.054 to 0.649)	.10	0.105 (−1.022 to 1.233)	.85
No. of comorbidities^j^	0.108 (0.061 to 0.156)	<.001	0.921 (0.773 to 1.070)	<.001
No. of NPTs ever used for chronic low back pain^k^	−0.035 (−0.087 to 0.016)	.18	0.065 (−0.113 to 0.244)	.47
Current opioid use for chronic low back pain				
No	[Reference]	<.001	[Reference]	<.001
Yes	0.351 (0.221 to 0.481)		1.205 (0.870 to 1.541)	
Prior lumbar spine surgery				
No	[Reference]	.67	[Reference]	.003
Yes	0.042 (−0.148 to 0.232)		0.992 (0.344 to 1.639)	

Abbreviations: CARE, Consultation and Relational Empathy measure; NA, not applicable; NPTs, nonpharmacological treatments; SEP, slightly empathic physician; VEP, very empathic physician.

^a^ Results are based on 1470 patients and 5943 encounters. Pain intensity, back-related disability, current opioid use for chronic low back pain, and prior lumbar spine surgery were measured at quarterly encounters over 12 months; all other characteristics were measured at baseline.

^b^ Pain intensity was measured with a numerical rating scale, ranging from 0 to 10, with higher scores indicating worse outcomes for pain.

^c^ β-Coefficients were derived from generalized estimating equations adjusted for all other characteristics in the table. They represent change in each outcome per unit increase in the respective measures or relative to the reference categories. Negative β-coefficients indicate better outcomes.

^d^ Back-related disability was measured with the Roland-Morris Disability Questionnaire, with scores ranging from 0 to 24 and higher scores indicating worse function.

^e^ Physicians were classified, based on CARE measure scores, as very empathic if their scores were 30 or higher and as slightly empathic if their scores were 29 or lower. Scores ranged from 10 to 50, with higher scores indicating greater physician empathy. Slightly empathic physicians made up approximately the lowest quartile on the CARE measure.

^f^ Results for the empathy × time interaction refer to patients having VEPs compared with those having SEPs.

^g^ Refers to increasing years of age. The β-coefficients represent change in the outcome variables (eg, pain intensity) with each additional year of age.

^h^ Racial categories other than White were combined because small numbers in some racial categories yielded imprecise estimates that precluded meaningful interpretation.

^i^ Educational level was scored as 1 to indicate no high school diploma; 2, high school graduate or high school equivalency diploma; 3, some college, no degree; 4, occupational, technical, or vocational program; 5, associate’s degree; 6, bachelor’s degree; 7, master’s degree; or 8, professional school degree or doctoral degree.

^j^ Range was from 0 to 9 for the number of comorbidities.

^k^ Range was from 0 to 6 for the number of NPTs ever used for chronic low back pain.

**Table 3.** Characteristics Associated With Health-Related Quality-of-Life Outcomes for 1470 Patients and 5943 Encounters Over Time^a^

Characteristic	Anxiety		Depression		Fatigue		Sleep disturbance		Pain interference	
	β (95% CI)^b^	*P* value	β (95% CI)^b^	*P* value	β (95% CI)^b^	*P* value	β (95% CI)^b^	*P* value	β (95% CI)^b^	*P* value
Physician type^c^										
SEP	[Reference]	<.001	[Reference]	<.001	[Reference]	<.001	[Reference]	<.001	[Reference]	<.001
VEP	−2.058 (−3.275 to −0.842)		−2.309 (−3.473 to −1.146)		−2.816 (−3.938 to −1.694)		−2.398 (−3.399 to −1.397)		−2.356 (−3.194 to −1.518)	
Quarterly intervals, No.	−0.042 (−0.309 to 0.225)	.76	−0.011 (−0.265 to 0.243)	.93	−0.032 (−0.272 to 0.208)	.79	−0.236 (−0.447 to −0.025)	.03	−0.273 (−0.468 to −0.077)	.006
Physician empathy group × time^d^	−0.077 (−0.377 to 0.223)	.62	−0.109 (−0.391 to 0.174)	.45	−0.095 (−0.369 to 0.178)	.50	0.102 (−0.138 to 0.341)	.41	−0.018 (−0.239 to 0.204)	.87
Age, y^e^	−0.154 (−0.189 to −0.118)	<.001	−0.123 (−0.156 to −0.089)	<.001	−0.135 (−0.169 to −0.100)	<.001	−0.103 (−0.133 to −0.073)	<.001	−0.003 (−0.027 to 0.022)	.84
Female	0.887 (−0.115 to 1.888)	.08	0.307 (−0.649 to 1.263)	.53	2.104 (1.106 to 3.102)	<.001	0.142 (−0.658 to 0.941)	.73	1.352 (0.594 to 2.111)	<.001
Race										
Non-White^f^	1.239 (0.136 to 2.342)	.03	−0.533 (−1.601 to 0.536)	.33	−1.088 (−2.224 to 0.048)	.06	−0.200 (−1.127 to 0.726)	.67	0.992 (0.167 to 1.816)	.02
White	[Reference]		[Reference]		[Reference]		[Reference]		[Reference]	
Ethnicity										
Hispanic	0.643 (−0.958 to 2.244)	.43	0.224 (−1.196 to 1.644)	.76	0.704 (−0.916 to 2.324)	.39	0.531 (−0.742 to 1.804)	.41	0.400 (−0.640 to 1.439)	.45
Non-Hispanic	[Reference]		[Reference]		[Reference]		[Reference]		[Reference]	
Educational level^g^	−0.575 (−0.812 to −0.338)	<.001	−0.721 (−0.950 to −0.493)	<.001	−0.586 (−0.820 to −0.352)	<.001	−0.562 (−0.767 to −0.356)	<.001	−0.769 (−0.938 to −0.600)	<.001
Smoking status										
Never or former	[Reference]	<.001	[Reference]	<.001	[Reference]	.34	[Reference]	.03	[Reference]	<.001
Current	2.376 (1.178 to 3.574)		2.073 (0.887 to 3.259)		0.588 (−0.611 to 1.788)		1.128 (0.125 to 2.130)		1.595 (0.739 to 2.451)	
Low back pain duration, y										
<1	[Reference]	NA	[Reference]	NA	[Reference]	NA	[Reference]	NA	[Reference]	NA
1-5	−0.349 (−2.186 to 1.487)	.71	−0.130 (−1.825 to 1.566)	.88	2.029 (0.123 to 3.936)	.04	0.933 (−0.591 to 2.458)	.23	0.354 (−0.914 to 1.623)	.58
>5	−0.694 (−2.436 to 1.049)	.44	−0.575 (−2.171 to 1.022)	.48	2.219 (0.405 to 4.033)	.02	0.589 (−0.851 to 2.029)	.42	0.745 (−0.458 to 1.949)	.22
Comorbidities, No.^h^	1.242 (0.981 to 1.503)	<.001	1.485 (1.244 to 1.727)	<.001	1.657 (1.411 to 1.902)	<.001	0.751 (0.536 to 0.966)	<.001	1.232 (1.052 to 1.412)	<.001
NPTs for chronic low back pain, No.^i^	0.534 (0.243 to 0.825)	<.001	0.216 (−0.058 to 0.490)	.12	0.328 (0.045 to 0.612)	.02	0.072 (−0.167 to 0.310)	.56	0.145 (−0.062 to 0.352)	.17
Current opioid use for chronic low back pain	0.969 (0.347 to 1.590)	.002	1.183 (0.576 to 1.791)	<.001	1.164 (0.539 to 1.789)	<.001	0.953 (0.425 to 1.481)	<.001	1.731 (1.230 to 2.232)	<.001
Prior lumbar spine surgery	−0.007 (−1.023 to 1.009)	.99	0.462 (−0.462 to 1.387)	.33	−0.179 (−1.111 to 0.754)	.71	1.480 (0.650 to 2.310)	<.001	0.873 (0.167 to 1.580)	.02

Abbreviations: CARE, Consultation and Relational Empathy measure; NA, not applicable; NPTs, nonpharmacological treatments; SEP, slightly empathic physician; VEP, very empathic physician.

^a^ Health-related quality-of-life outcomes were measured at quarterly encounters over 12 months using the Patient-Reported Outcomes Measurement Information System. Each deficit measure included 4 ordinal-scale items scored and normed per the US general population to yield mean (SD) scores of 50 (10). The exception was sleep disturbance, which was normed using a calibration sample enriched with patients with chronic illness. Higher scores indicate worse outcomes. Current opioid use for chronic low back pain and prior lumbar spine surgery was also measured at quarterly encounters; all other characteristics were measured at baseline.

^b^ β-Coefficients were derived from generalized estimating equations adjusted for all other characteristics and represent change in each outcome per unit increase in the measures or relative to the reference categories. Negative β-coefficients indicate better outcomes.

^c^ Physicians were classified based on CARE measure scores as very empathic (≥30) or slightly empathic (≤29). Slightly empathic physicians made up approximately the lowest quartile on the CARE measure.

^d^ Patients with VEPs vs with those with SEPs.

^e^ Refers to increasing years of age. The β-coefficients represent change in the outcome variables (eg, anxiety) with each additional year of age.

^f^ Racial categories other than White were combined because small numbers in some racial categories yielded imprecise estimates that precluded meaningful interpretation.

^g^ Scored as 1, no high school diploma; 2, high school graduate or equivalency diploma; 3, some college, no degree; 4, occupational, technical, or vocational program; 5, associate’s degree; 6, bachelor’s degree; 7, master’s degree; or 8, professional school degree or doctoral degree.

^h^ Range was from 0 to 9.

^i^ Range was from 0 to 6.

## Discussion

In this cohort study of adults with CLBP, physician empathy was inversely associated with pain intensity, back-related disability, and HRQOL deficits in all main analyses, including those that controlled for time effects and a wide array of sociodemographic and clinical covariates at baseline and also for current opioid use and lumbar spine surgery over 12 months. Moreover, all VEP compared with SEP group differences were clinically relevant. Similar trends were observed in sensitivity analyses that measured outcomes after altering cut points for classifying physician empathy. Empathy is an essential aspect of the patient-physician relationship in delivering patient-centered care. It is particularly important in pain medicine in which traditional, hard outcome measures are not often available, and softer outcomes driven by patient perceptions of pain, function, and HRQOL are generally the rule. Patients who somatize their chronic pain may be more likely to discuss psychosocial issues with VEPs, thereby directing diagnostic and therapeutic efforts down more rewarding paths that enhance compliance and outcomes.^25^

Although our findings suggest that greater physician empathy should be encouraged during encounters for chronic pain, there is a longstanding debate about whether it can or should be taught.^26^ One view is that physician empathy cannot be achieved in the patient-physician relationship and questions attempts to measure it.^27^ An alternative view is that it is a skill that, although partly genetic, can be purposefully grown, broadened, and fine-tuned through life experiences to improve medical care.^28^ An aspirational view is that empathy should not be limited to individual practitioners but should be incorporated within the broader domain of health care systems.^29^ Such systems would be structured and organized to facilitate empathic health care delivery using macro-level decisions involving political and jurisdictional considerations.

Medical students and residents often become less empathic during education and training, owing to a greater perceived need for patient detachment and reliance on technology. The challenges of contemporary medicine, including electronic medical records and time constraints, may also contribute to an erosion of empathy among seasoned physicians.^30^ Although more research is needed on interventions to cultivate physician empathy,^31^ a randomized clinical trial of training grounded in the neurobiology of empathy demonstrated significant improvements in patient-reported empathy among resident physicians.^32^ Our sensitivity analysis findings suggest that the greatest improvements in pain-related outcomes may be achieved by targeting for intervention those physicians comprising the lowest decile of empathy. More research involving rigorous designs is needed to determine if greater physician empathy improves clinical outcomes.

### Strengths and Limitations

Strengths of our study include recruiting participants through a national registry, longitudinal follow-up for 12 months, and multivariable adjustment of outcomes for potential confounders. We also measured empathy as perceived by patients rather than self-reported by physicians. By contrast, a systematic review of interventions to cultivate physician empathy found that 58 of 64 studies, including most of the least rigorous studies, used physician self-reported measures of empathy.^31^ Finally, we only included patients who retained the same physician during all encounters, and CARE measure scores among registry participants with 24 months of follow-up indicated that physician empathy remained remarkably stable over time.

There are also limitations of this study. First, although patients resembled adults with CLBP in the National Health and Nutrition Examination Survey on characteristics such as age, sex, educational level, cigarette smoking status, and medical comorbidities,^33^ PRECISION is not a population-based registry, and results may be subject to participant volunteer bias. Moreover, we excluded participants who were unable to complete case report forms in English. Second, although patients included only those with a physician who provided CLBP usual care, factors such as physician demographic characteristics, racial concordance, specialty, and clinical setting were not measured. Third, the registry did not collect sensitive participant data such as income or health insurance coverage. Thus, we relied on educational level as a surrogate measure for socioeconomic factors in our models with covariate adjustment. Fourth, we cannot rule out that patient ratings of physician empathy at enrollment may have been affected by preexisting pain, function, or HRQOL. However, adjusting for baseline values of these outcome variables would have negated prior benefits in these domains that may have been attributable to physician empathy. Finally, all data pertaining to explanatory and outcome variables were self-reported and not otherwise corroborated.

## Conclusions

In this cohort study of patients with chronic pain, physician empathy was associated with better longitudinal outcomes in pain, function, and HRQOL over 12 months, including in multivariable analyses that controlled for time effects and a comprehensive array of covariates that included current opioid use and lumbar spine surgery throughout the study. The outcomes of having a VEP were clinically relevant and better than those associated with nonpharmacological treatments, opioid therapy, and lumbar spine surgery. Physician empathy is an important aspect of the patient-physician relationship among those with chronic pain. Greater efforts to cultivate and improve physician empathy appear warranted in this population.

## Notes

Supplement 1. **eTable 1.** Outcomes Based on Physician Empathy (CARE Measure Score), Time, and Empathy × Time Interaction **eTable 2.** Clinical Relevance of Outcomes Based on Physician Empathy Group (VEP vs SEP), Time, and Empathy × Time Interaction **eTable 3.** Outcomes Based on Physician Empathy (CARE Measure Score), Time, Empathy × Time Interaction, and the Full Array of Covariates **eTable 4.** Clinical Relevance of Outcomes Based on Physician Empathy Group (VEP vs SEP), Time, Empathy × Time Interaction, and the Full Array of Covariates **eTable 5.** Clinical Relevance of Outcomes Based on Physician Empathy Group (Greater vs Lesser Physician Empathy), Time, and Empathy × Time Interaction According to Alternative Cut Points for Physician Empathy **eTable 6.** Clinical Relevance of Outcomes Based on Physician Empathy Group (Greater vs Lesser Physician Empathy), Time, Empathy × Time Interaction, and the Full Array of Covariates According to Alternative Cut Points for Physician Empathy

Supplement 2.
Data Sharing Statement
