# Supplementary material for: Physician Empathy and Chronic Pain Outcomes
Source: JAMA Netw Open. 2024 Apr 11;7(4):e246026. doi: 10.1001/jamanetworkopen.2024.6026 (PMC11009829; doi:10.1001/jamanetworkopen.2024.6026)
Supplement: Supplement 1. — eTable 1. Outcomes Based on Physician Empathy (CARE Measure Score), Time, and Empathy × Time Interaction eTable 2. Clinical Relevance of Outcomes Based on Physician Empathy Group (VEP vs SEP), Time, and Empathy × Time Interaction eTable 3. Outcomes Based on Physician Empathy (CARE Measure Score), Time, Empathy × Time Interaction, and the Full Array of Covariates eTable 4. Clinical Relevance of Outcomes Based on Physician Empathy Group (VEP vs SEP), Time, Empathy × Time Interaction, and the Full Array of Covariates eTable 5. Clinical Relevance of Outcomes Based on Physician Empathy Group (Greater vs Lesser Physician Empathy), Time, and Empathy × Time Interaction According to Alternative Cut Points for Physician Empathy eTable 6. Clinical Relevance of Outcomes Based on Physician Empathy Group (Greater vs Lesser Physician Empathy), Time, Empathy × Time Interaction, and the Full Array of Covariates According to Alternative Cut Points for Physician Empathy [file jamanetwopen-e246026-s001.pdf]

Supplemental Online Content

Licciardone JC, Tran Y, Ngo K, Toledo D, Peddireddy N, Aryal S. Physician empathy and chronic pain outcomes. *JAMA Netw Open*. 2024;7(4):e246026. doi:10.1001/jamanetworkopen.2024.6026

eTable 1. Outcomes Based on Physician Empathy (CARE Measure Score), Time, and Empathy × Time Interaction
eTable 2. Clinical Relevance of Outcomes Based on Physician Empathy Group (VEP vs SEP), Time, and Empathy × Time Interaction
eTable 3. Outcomes Based on Physician Empathy (CARE Measure Score), Time, Empathy × Time Interaction, and the Full Array of Covariates
eTable 4. Clinical Relevance of Outcomes Based on Physician Empathy Group (VEP vs SEP), Time, Empathy × Time Interaction, and the Full Array of Covariates
eTable 5. Clinical Relevance of Outcomes Based on Physician Empathy Group (Greater vs Lesser Physician Empathy), Time, and Empathy × Time Interaction According to Alternative Cut Points for Physician Empathy
eTable 6. Clinical Relevance of Outcomes Based on Physician Empathy Group (Greater vs Lesser Physician Empathy), Time, Empathy × Time Interaction, and the Full Array of Covariates According to Alternative Cut Points for Physician Empathy

This supplemental material has been provided by the authors to give readers additional information about their work.

© 2024 Licciardone JC et al. *JAMA Network Open*.

eTable 1. Outcomes Based on Physician Empathy (CARE Measure Score), Time, and Empathy × Time Interaction[a]

Outcome	β (95% CI)	*P*		
		Physician Empathy	Time	Empathy x Time
Pain intensity	−0.013 (−0.022 to −0.005)	0.003	0.04	0.91
Back-related disability	−0.066 (−0.091 to −0.040)	<0.001	0.77	0.38
Anxiety	−0.081 (−0.129 to −0.034)	<0.001	0.25	0.07
Depression	−0.106 (−0.150 to −0.062)	<0.001	0.46	0.19
Fatigue	−0.107 (−0.152 to −0.061)	<0.001	0.31	0.11
Sleep disturbance	−0.088 (−0.127 to −0.049)	<0.001	0.38	0.96
Pain interference	−0.084 (−0.118 to −0.049)	<0.001	0.14	0.80

[a]Results are based on 1470 patients and 5943 encounters. Pain intensity was measured with a numerical rating scale, ranging from 0 to 10. Back-related disability was measured with the Roland-Morris Disability Questionnaire, ranging from 0 to 24. The five health-related quality-of-life outcomes were measured using the Patient-Reported Outcomes Measurement Information System with 29 items, with scores generally ranging from 40 to 70 on each scale. β-coefficients were derived from generalized estimating equations and represent changes in each outcome per unit increase in the CARE measure score for physician empathy. Negative β-coefficients represent improvements in each outcome with increasing physician empathy. CARE denotes Consultation and Relational Empathy.

© 2024 Licciardone JC et al. *JAMA Network Open*.

eTable 2. Clinical Relevance of Outcomes Based on Physician Empathy Group (VEP vs SEP), Time, and Empathy × Time Interaction[a]

	VEP	SEP		*P*		
**Outcome**	**Mean (95% CI)**	**Mean (95% CI)**	**Cohen *d***	**Physician Empathy**	**Time**	**Empathy x Time**
Pain intensity	5.7 (5.6-5.8)	6.2 (6.0-6.3)	0.22	0.002	0.004	0.43
Back-related disability	13.5 (13.1-13.8)	15.6 (15.0-16.1)	0.33	<0.001	0.40	0.22
Anxiety	54.6 (54.1-55.1)	57.5 (56.5-58.4)	0.28	<0.001	0.83	0.44
Depression	53.0 (52.5-53.5)	56.1 (55.2-57.1)	0.33	<0.001	0.96	0.32
Fatigue	58.0 (57.4-58.5)	61.6 (60.7-62.5)	0.36	<0.001	0.96	0.35
Sleep disturbance	56.6 (56.2-57.0)	59.1 (58.3-59.8)	0.30	<0.001	0.05	0.55
Pain interference	61.9 (61.5-62.3)	64.6 (63.9-65.3)	0.34	<0.001	0.01	0.68

[a]Results are based on 1470 patients and 5943 encounters. The VEP group was comprised of patients having a physician with a Consultation and Relational Empathy measure score≥30, whereas the SEP group was comprised of patients having a physician with a Consultation and Relational Empathy measure score≤29. Pain intensity was measured with a numerical rating scale, ranging from 0 to 10. Back-related disability was measured with the Roland- Morris Disability Questionnaire, ranging from 0 to 24. The five health-related quality-of-life outcomes were measured using the Patient-Reported Outcomes Measurement Information System with 29 items, with scores generally ranging from 40 to 70 on each scale. Mean outcomes were derived from generalized estimating equations. Lower means represent better outcomes on each measure. Cohen *d* statistics ≥0.20 represent a clinically relevant difference between physician empathy groups. SEP denotes slightly empathic physician; VEP, very empathic physician.

© 2024 Licciardone JC et al. *JAMA Network Open*.

eTable 3. Outcomes Based on Physician Empathy (CARE Measure Score), Time, Empathy × Time Interaction, and the Full Array of Covariates[a]

		P		
**Outcome**	**β (95% CI)**	**Physician Empathy**	**Time**	**Empathy x Time**
Pain intensity	−0.014 (−0.022 to −0.006)	<0.001	0.02	0.73
Back-related disability	−0.062 (−0.085 to −0.040)	<0.001	0.50	0.64
Anxiety	−0.067 (−0.112 to −0.022)	0.004	0.36	0.15
Depression	−0.091 (−0.133 to −0.049)	<0.001	0.65	0.34
Fatigue	−0.094 (−0.136 to −0.051)	<0.001	0.48	0.20
Sleep disturbance	−0.079 (−0.116 to −0.042)	<0.001	0.22	0.70
Pain interference	−0.080 (−0.111 to −0.049)	<0.001	0.07	0.93

[a]Results are based on 1470 patients and 5943 encounters. Pain intensity was measured with a numerical rating scale, ranging from 0 to 10. Back-related disability was measured with the Roland-Morris Disability Questionnaire, ranging from 0 to 24. The five health-related quality-of-life outcomes were measured using the Patient-Reported Outcomes Measurement Information System with 29 items, with scores generally ranging from 40 to 70 on each scale. β-coefficients were derived from generalized estimating equations including the full array of covariates shown in Table 2 and represent changes in each outcome per unit increase in the CARE measure score for physician empathy. Negative β-coefficients represent improvements in each outcome with increasing physician empathy. CARE denotes Consultation and Relational Empathy.

© 2024 Licciardone JC et al. *JAMA Network Open*.

eTable 4. Clinical Relevance of Outcomes Based on Physician Empathy Group (VEP vs SEP), Time, Empathy × Time Interaction, and the Full Array of Covariates[a]

	VEP Group	SEP Group		*P*		
**Outcome**	**Mean (95% CI)**	**Mean (95% CI)**	**Cohen *d***	**Physician Empathy**	**Time**	**Empathy x Time**
Pain intensity	6.3 (6.1-6.5)	6.7 (6.5-6.9)	0.21	<0.001	0.003	0.68
Back-related disability	14.9 (14.2-15.6)	16.8 (16.0-17.6)	0.30	<0.001	0.26	0.36
Anxiety	56.3 (55.2-57.5)	58.6 (57.3-60.0)	0.22	<0.001	0.76	0.62
Depression	54.2 (53.1-55.3)	56.8 (55.5-58.0)	0.27	<0.001	0.93	0.45
Fatigue	57.3 (56.1-58.5)	60.4 (59.0-61.7)	0.30	<0.001	0.79	0.50
Sleep disturbance	57.6 (56.6-58.6)	59.7 (58.6-60.9)	0.25	<0.001	0.03	0.41
Pain interference	62.9 (62.1-63.7)	65.3 (64.4-66.3)	0.30	<0.001	0.006	0.87

[a]Results are based on 1470 patients and 5943 encounters. The VEP group was comprised of patients having a physician with a Consultation and Relational Empathy measure score≥30, whereas the SEP group was comprised of patients having a physician with a Consultation and Relational Empathy measure score≤29. Pain intensity was measured with a numerical rating scale, ranging from 0 to 10. Back-related disability was measured with the Roland- Morris Disability Questionnaire, ranging from 0 to 24. The five health-related quality-of-life outcomes were measured using the Patient-Reported Outcomes Measurement Information System with 29 items, with scores generally ranging from 40 to 70 on each scale. Mean outcomes were derived from generalized estimating equations including the full array of covariates shown in Table 2. Lower means represent better outcomes on each measure. Cohen *d* statistics ≥0.20 represent a clinically relevant difference between physician empathy groups. SEP denotes slightly empathic physician; VEP, very empathic physician.

© 2024 Licciardone JC et al. *JAMA Network Open*.

eTable 5. Clinical Relevance of Outcomes Based on Physician Empathy Group (Greater vs Lesser Physician Empathy), Time, and Empathy × Time Interaction According to Alternative Cut Points for Physician Empathy[a]

Outcome	Greater Physician Empathy Mean (95% CI)	Lesser Physician Empathy Mean (95% CI)	Cohen *d*	*P* Physician Empathy	*P* Time	*P* Empathy x Time
Pain intensity						
CARE=50 vs. CARE≤49	5.9 (5.7-6.1)	5.8 (5.7-5.9)	−0.05	0.39	<0.001	0.93
CARE≥40 vs. CARE≤39	5.7 (5.6-5.9)	5.9 (5.8-6.1)	0.10	0.06	<0.001	0.95
CARE≥30 vs. CARE≤29	5.7 (5.6-5.8)	6.2 (6.0-6.3)	0.22	0.002	0.004	0.43
CARE≥20 vs. CARE≤19	5.8 (5.7-5.9)	6.4 (6.1-6.6)	0.29	<0.001	0.006	0.57
Back-related disability						
CARE=50 vs. CARE≤49	13.6 (13.0-14.2)	14.1 (13.7-14.4)	0.07	0.24	0.001	0.98
CARE≥40 vs. CARE≤39	13.5 (13.1-13.9)	14.5 (14.1-15.0)	0.17	<0.001	0.005	0.68
CARE≥30 vs. CARE≤29	13.5 (13.1-13.8)	15.6 (15.0-16.1)	0.33	<0.001	0.40	0.22
CARE≥20 vs. CARE≤19	13.7 (13.4-14.0)	16.7 (15.9-17.6)	0.47	<0.001	0.72	0.38
Anxiety						
CARE=50 vs. CARE≤49	53.3 (52.3-54.3)	56.0 (55.4-56.5)	0.27	<0.001	0.21	0.31
CARE≥40 vs. CARE≤39	54.5 (53.9-55.1)	56.3 (55.6-57.0)	0.17	0.15	0.44	0.007
CARE≥30 vs. CARE≤29	54.6 (54.1-55.1)	57.5 (56.5-58.4)	0.28	<0.001	0.83	0.44
CARE≥20 vs. CARE≤19	55.0 (54.5-55.4)	58.6 (57.0-60.1)	0.36	<0.001	0.85	0.71
Depression						
CARE=50 vs. CARE≤49	51.7 (50.7-52.6)	54.4 (53.9-54.9)	0.28	<0.001	0.02	0.18
CARE≥40 vs. CARE≤39	52.8 (52.2-53.4)	54.9 (54.3-55.6)	0.22	0.002	0.97	0.12
CARE≥30 vs. CARE≤29	53.0 (52.5-53.5)	56.1 (55.2-57.1)	0.33	<0.001	0.96	0.32
CARE≥20 vs. CARE≤19	53.4 (52.9-53.8)	57.3 (55.8-58.8)	0.41	<0.001	0.75	0.38
Fatigue						
CARE=50 vs. CARE≤49	57.6 (56.6-58.7)	59.2 (58.7-59.7)	0.15	0.07	0.22	0.40
CARE≥40 vs. CARE≤39	57.9 (57.3-58.5)	60.0 (59.3-60.7)	0.21	0.004	0.97	0.11
CARE≥30 vs. CARE≤29	58.0 (57.4-58.5)	61.6 (60.7-62.5)	0.36	<0.001	0.96	0.35
CARE≥20 vs. CARE≤19	58.3 (57.8-58.8)	64.0 (62.6-65.5)	0.56	<0.001	0.59	0.25
Sleep disturbance						
CARE=50 vs. CARE≤49	56.5 (55.7-57.4)	57.4 (57.0-57.8)	0.10	0.19	0.01	0.73
CARE≥40 vs. CARE≤39	56.6 (56.1-57.2)	57.9 (57.3-58.4)	0.15	0.01	0.08	0.73
CARE≥30 vs. CARE≤29	56.6 (56.2-57.0)	59.1 (58.3-59.8)	0.30	<0.001	0.05	0.55
CARE≥20 vs. CARE≤19	56.8 (56.4-57.2)	60.9 (59.7-62.1)	0.49	<0.001	0.11	0.45
Pain interference						
CARE=50 vs. CARE≤49	62.0 (61.2-62.8)	62.7 (62.3-63.1)	0.09	0.18	<0.001	0.95
CARE≥40 vs. CARE≤39	62.0 (61.5-62.5)	63.1 (62.6-63.6)	0.14	0.01	<0.001	0.85
CARE≥30 vs. CARE≤29	61.9 (61.5-62.3)	64.6 (63.9-65.3)	0.34	<0.001	0.01	0.68
CARE≥20 vs. CARE≤19	62.2 (61.8-62.6)	66.0 (64.9-67.1)	0.49	<0.001	0.001	0.18

© 2024 Licciardone JC et al. *JAMA Network Open*.

[a]Results are based on 1470 patients and 5943 encounters. The number of patients in the greater physician empathy groups were 374 (25.5%), 836 (56.9%), 1133 (77.1%), and 1348 (91.7%) using CARE=50, CARE≥40, CARE≥30, and CARE≥20, respectively, as cut points. Pain intensity was measured with a numerical rating scale, ranging from 0 to 10. Back-related disability was measured with the Roland-Morris Disability Questionnaire, ranging from 0 to 24. The five health-related quality-of-life outcomes were measured using the Patient-Reported Outcomes Measurement Information System with 29 items, with scores generally ranging from 40 to 70 on each scale. Mean outcomes were derived from generalized estimating equations. Lower means represent better outcomes on each measure. Cohen *d* statistics ≥0.20 represent a clinically relevant difference between physician empathy groups. Results for CARE≥30 vs. CARE≤29 represent the main analyses, whereas the remaining contrasts represent the sensitivity analyses. CARE denotes the Consultation and Relational Empathy measure score.

© 2024 Licciardone JC et al. *JAMA Network Open*.

eTable 6. Clinical Relevance of Outcomes Based on Physician Empathy Group (Greater vs Lesser Physician Empathy), Time, Empathy × Time Interaction, and the Full Array of Covariates According to Alternative Cut Points for Physician Empathy[a]

Outcome	Greater Physician Empathy Mean (95% CI)	Lesser Physician Empathy Mean (95% CI)	Cohen *d*	*P* Physician Empathy	*P* Time	*P* Empathy x Time
Pain intensity						
CARE=50 vs. CARE≤49	6.4 (6.1-6.6)	6.4 (6.2-6.6)	−0.01	0.99	<0.001	0.77
CARE≥40 vs. CARE≤39	6.3 (6.1-6.5)	6.5 (6.3-6.7)	0.10	0.02	<0.001	0.71
CARE≥30 vs. CARE≤29	6.3 (6.1-6.5)	6.7 (6.5-6.9)	0.21	<0.001	0.003	0.68
CARE≥20 vs. CARE≤19	6.3 (6.1-6.5)	6.8 (6.5-7.2)	0.26	<0.001	0.01	0.63
Back-related disability						
CARE=50 vs. CARE≤49	15.0 (14.2-15.9)	15.5 (14.8-16.2)	0.07	0.13	<0.001	0.85
CARE≥40 vs. CARE≤39	14.9 (14.1-15.6)	15.9 (15.2-16.7)	0.16	<0.001	0.002	0.46
CARE≥30 vs. CARE≤29	14.9 (14.2-15.6)	16.8 (16.0-17.6)	0.30	<0.001	0.26	0.36
CARE≥20 vs. CARE≤19	15.1 (14.4-15.8)	17.6 (16.6-18.7)	0.39	<0.001	0.65	0.45
Anxiety						
CARE=50 vs. CARE≤49	55.2 (53.8-56.6)	57.6 (56.4-58.7)	0.24	<0.001	0.26	0.53
CARE≥40 vs. CARE≤39	56.2 (55.0-57.4)	57.7 (56.5-59.0)	0.15	0.21	0.44	0.02
CARE≥30 vs. CARE≤29	56.3 (55.2-57.5)	58.6 (57.3-60.0)	0.22	<0.001	0.76	0.62
CARE≥20 vs. CARE≤19	56.7 (55.5-57.8)	59.1 (57.3-60.9)	0.24	0.02	0.81	0.83
Depression						
CARE=50 vs. CARE≤49	53.2 (51.8-54.5)	55.5 (54.4-56.6)	0.23	<0.001	0.02	0.09
CARE≥40 vs. CARE≤39	53.9 (52.8-55.1)	55.9 (54.7-57.0)	0.20	0.002	0.93	0.21
CARE≥30 vs. CARE≤29	54.2 (53.1-55.3)	56.8 (55.5-58.0)	0.27	<0.001	0.93	0.45
CARE≥20 vs. CARE≤19	54.6 (53.5-55.6)	57.4 (55.7-59.2)	0.29	0.005	0.78	0.44
Fatigue						
CARE=50 vs. CARE≤49	57.3 (55.8-58.7)	58.3 (57.2-59.5)	0.11	0.16	0.18	0.59
CARE≥40 vs. CARE≤39	57.1 (55.9-58.4)	59.1 (57.9-60.4)	0.20	0.002	0.86	0.20
CARE≥30 vs. CARE≤29	57.3 (56.1-58.5)	60.4 (59.0-61.7)	0.30	<0.001	0.79	0.50
CARE≥20 vs. CARE≤19	57.6 (56.4-58.8)	62.2 (60.4-64.0)	0.46	<0.001	0.67	0.31
Sleep disturbance						
CARE=50 vs. CARE≤49	57.7 (56.5-58.9)	58.3 (57.3-59.3)	0.07	0.27	0.006	0.99
CARE≥40 vs. CARE≤39	57.6 (56.6-58.7)	58.7 (57.7-59.8)	0.13	0.01	0.05	0.98
CARE≥30 vs. CARE≤29	57.6 (56.6-58.6)	59.7 (58.6-60.9)	0.25	<0.001	0.03	0.41
CARE≥20 vs. CARE≤19	57.8 (56.9-58.8)	61.2 (59.7-62.6)	0.40	<0.001	0.10	0.41
Pain interference						
CARE=50 vs. CARE≤49	63.1 (62.1-64.1)	63.7 (62.9-64.5)	0.08	0.14	<0.001	0.84
CARE≥40 vs. CARE≤39	63.0 (62.2-63.9)	64.1 (63.3-65.0)	0.14	0.002	<0.001	0.88
CARE≥30 vs. CARE≤29	62.9 (62.1-63.7)	65.3 (64.4-66.3)	0.30	<0.001	0.006	0.87
CARE≥20 vs. CARE≤19	63.2 (62.4-64.0)	66.5 (65.2-67.7)	0.41	<0.001	0.001	0.20

© 2024 Licciardone JC et al. *JAMA Network Open*.

[a]Results are based on 1470 patients and 5943 encounters. The number of patients in the greater physician empathy groups were 374 (25.5%), 836 (56.9%), 1133 (77.1%), and 1348 (91.7%) using CARE=50, CARE≥40, CARE≥30, and CARE≥20, respectively, as cut points. Pain intensity was measured with a numerical rating scale, ranging from 0 to 10. Back-related disability was measured with the Roland-Morris Disability Questionnaire, ranging from 0 to 24. The five health-related quality-of-life outcomes were measured using the Patient-Reported Outcomes Measurement Information System with 29 items, with scores generally ranging from 40 to 70 on each scale. Mean outcomes were derived from generalized estimating equations. Lower means represent better outcomes on each measure. Cohen *d* statistics ≥0.20 represent a clinically relevant difference between physician empathy groups. Results for CARE≥30 vs. CARE≤29 represent the main analyses, whereas the remaining contrasts represent the sensitivity analyses. CARE denotes the Consultation and Relational Empathy measure score.

© 2024 Licciardone JC et al. *JAMA Network Open*.
