# Supplementary material for: Physician Empathy and Chronic Pain Outcomes
Source: JAMA Netw Open. 2024 Apr 11;7(4):e246026. doi: 10.1001/jamanetworkopen.2024.6026 (PMC11009829; doi:10.1001/jamanetworkopen.2024.6026)
Supplement: Supplement 2. — Data Sharing Statement [file jamanetwopen-e246026-s002.pdf]

Data Sharing Statement

Licciardone. Physician Empathy and Chronic Pain Outcomes. *JAMA Netw Open*. Published April 11, 2024. doi:10.1001/jamanetworkopen.2024.6026

Data
Data available: No

Additional Information
Explanation for why data not available: These data belong to an existing registry that continues to use them to conduct ongoing research. Updates regarding the registry's future plans to share individual participant data may be posted to ClinicalTrials.gov.
